# Dataset for a case report of a homozygous PEX16 F332del mutation

**DOI:** 10.1016/j.dib.2015.12.011

**Published:** 2015-12-17

**Authors:** Carlos Bacino, Yu-Hsin Chao, Elaine Seto, Tim Lotze, Fan Xia, Richard O. Jones, Ann Moser, Michael F. Wangler

**Affiliations:** a Department of Molecular and Human Genetics, BCM, Houston, TX 77030, USA; b Department of Pediatrics, Division of Pediatric Neurology and Developmental Neuroscience, BCM, Houston, TX, USA; c Texas Children׳s Hospital, Houston, TX, USA; d Kennedy Krieger Institute, Baltimore, MD, USA

## Abstract

This dataset provides a clinical description along with extensive biochemical and molecular characterization of a patient with a homozygous mutation in PEX16 with an atypical phenotype. This patient described in Molecular Genetics and Metabolism Reports was ultimately diagnosed with an atypical peroxisomal disorder on exome sequencing. A clinical timeline and diagnostic summary, results of an extensive plasma and fibroblast analysis of this patient׳s peroxisomal profile is provided. In addition, a table of additional variants from the exome analysis is provided.

**Specifications Table**

**Table:** 

Subject area	*Genomics*
More specific subject area	*Peroxisomal Disorders*
Type of data	Table 1*– Biochemical analytes*
Table 2 – *Variants from exome sequencing*
Fig. 1 – *Clinical timeline and diagnostic workup*
Fig. 2*– Biochemical analytes*
How data was acquired	*Blood samples and a skin biopsy were obtained from the patient. DNA, plasma and cultured fibroblasts were analyzed in the context of the patient’s diagnostic course. LC–MS/MS, Enzyme activity using radioactive substrates, colorimetric assays, next-generation sequencing.*
Data format	*Analyzed datasets, Excel, Tif files.*
Experimental factors	*Unique genotype* (*n=1*)
Experimental features	*Plasma samples and cultured fibroblast from a skin biopsy were used for peroxisomal biochemical analysis. Genomic DNA was utilized for whole-exome sequencing.*
Data source location	*Houston Texas*
Data accessibility	*Date is included with this article*

**Value of the data**

• A profile of a patient with an atypical peroxisomal biogenesis disorder which can be compared with other patient׳s with these phenotypes.
• Clinical review of diagnostic considerations for atypical peroxisomal biogenesis disorders.
• Comprehensive set of functional consequences of F332del allele of *PEX16*.

## 1 Data

See Table 1.

1. Plasma VLCFA – plasma was collected at ages 10 years, 11 years and 22 years for VLCFA analysis. Values shown in ug/ml. C24/C22 and C26/C22 ratios shown. Z-scores of the patient׳s sample measurment as compared to a set of normal controls shown.
2. Fibroblast VLCFA – patient fibroblasts were cultured and analyzed for VLCFA analysis. Values shown are in µg/mg protein. Z-scores of the patient׳s sample measurment as compared to a set of normal controls shown.
3. Catalase Distribution – cultured cells were analyzed for Catalase Distribution (expressed in % soluble). A Z-score of the patient׳s sample is shown.
4. Plasmalogen synthesis assay from radiolabel enzyme assay is shown.
5. Plasma pipecolic acid (expressed in µmole/L).
6. Lyso-PC – LC MS/MS of lysophospholipids for the patient׳s blood sample at 22 years.
7. 14C oxidation assays for Phytanic and Pristanic acid (in % of the mean of controls) shown.

**Fig. 1:**
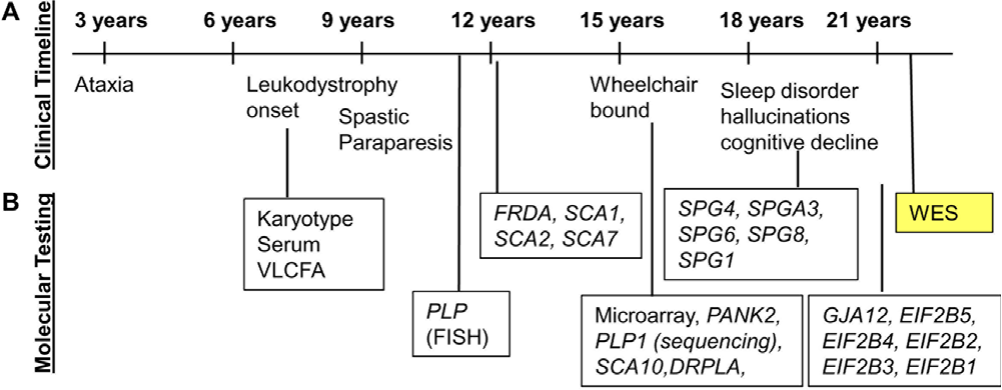
Clinical timeline for the patient.

**Fig. 2:**
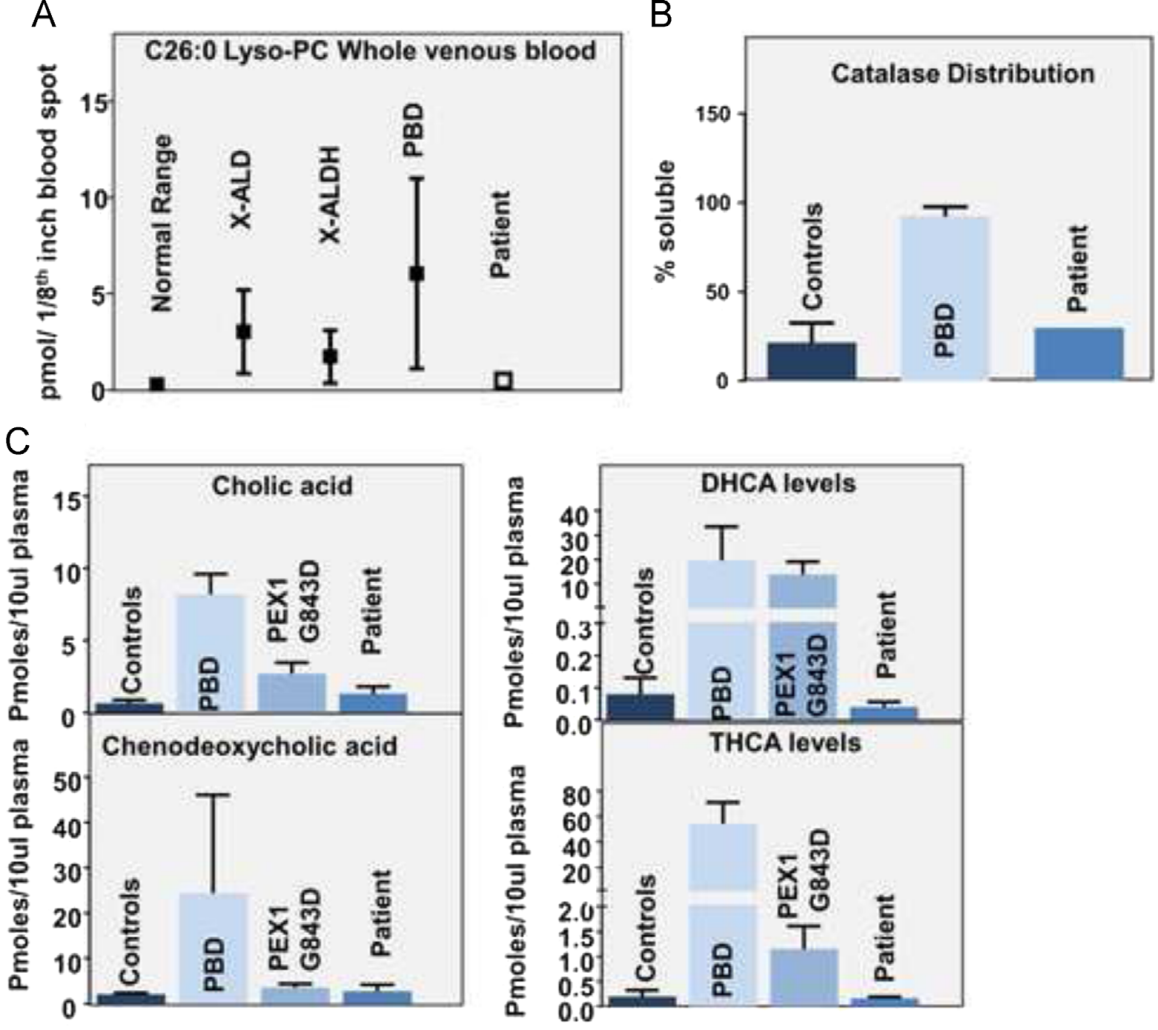
Peroxisomal biochemical studies. (A) C26:0 Lyso PC measured by LC–MS–MS for the Patient׳s plasma compared to Normals and other disease populations. (B) Catalase Distributionin cultured fibroblasts (expressed as % soluble). (C) Bile acid measurements in pmoles/10µl plasma for the Patient, controls and other disease populations.

List of variants in disease-causing genes including heterozygous and homozygous variants which were verified by Sanger sequencing. The Gene, position, specific isoform, nucleotide, protein change (predicted), and zygosity are shown. AR=Autosomal recessive. Comments contain segregation information from the parents or other populations (Table 2). A clinical and diagnostic timeline for the patient showing clinical events and gene diagnostic tests. WES=Whole-exome sequencing (Fig. 1, Fig. 2).

## 2 Experimental design, materials and methods

### 2.1 Ethics statement

Informed consent for the research and for publication was obtained prior to participation for the subject who was recruited under an Institutional Review Board approved protocol at Baylor College of Medicine.

### 2.2 Peroxisomal biochemical studies

Plasma samples and cultured fibroblast from a skin biopsy were used for peroxisomal biochemical analysis.

– Plasma pipecolic acid was measured by electron capture negative ion mass fragmentography [1].
– Very-long-chain fatty acid levels and total lipid fatty acid profile were measured as described [2], [3].
– The plasmalogen assay was performed using C14 radioactivity incorporation and H3 counts to measure microsomal plasmalogen steps [4].
– Fibroblast oxidation assays were performed using radioactive substrates to assay enzyme activity [5], [6].
– Measurement of C26:0-lyso-PC was performed as described [7] and bile acid quantitation was performed by tandem mass spectrometry [8].
– Catalase distribution in cultured cells was performed and quantified (% soluble catalase) [9], [10].

### 2.3 Whole-exome capture, sequencing and data analysis

The patient underwent WES through the Whole Genome Laboratory (https://www.bcm.edu/research/medical-genetics-labs/index.cfm?PMID=21319) using methods described [11].

– Produced sequence reads were aligned to the GRCh37 (hg19) human genome reference assembly using the HGSC Mercury analysis pipeline (http://www.tinyurl.com/HGSC-Mercury/). Variants were determined and called using the Atlas2 [12] suite to produce a variant call file (VCF [13]).
– High-quality variants were annotated using an in-house developed suite of annotation tools [14].

## Figures and Tables

**Table 1:** Comprehensive plasma and fibroblast biochemical analysis.

**Analyte**	**Control fibroblasts**	**Zellweger syndrome**	**Patient@22 years**
Phytanic acid oxidation (% mean of control value)	100	5.7	73
Pristanic acid oxication (% mean of control Value)	100	4.9	156.9

**Table 2:** Candidate variants table from Whole-exome sequencing.

**Gene**	**Postion**	**Isoform**	**Nucleotide**	**Protein**	**Zygosity**	**Disease**	**Disease inheritance**	**Comment**
CTC1	Ch17:8134658	NM_025099	c.2605C>T	p.Q869X	Het	Cerebroretinal microangiopathy with calcifications and cysts	AR	Father also heterozygous
SYNE1	Chr6:152730222	NM_033071	c.6542C>T	p.T2181I	Het	Spinocerebellar ataxia, autosomal recessive 8	AR	Mother also heterozygous
C5orf42	Chr5: 37185062	NM_023073	c.4309A>G	p.I1437V	Het	Joubert syndrome	AR	Novel variant
CLN3	CH16:28493901	uc010vcx.1	c.583C>G	p.P195A	Het	Ceroid lipofuscinosis	AR	rs146839771
VPS13A	Chr9:79902873	NM_033305	c.3356G>A	p.G1119E	Het	Choreoacanthocytosis	AR	rs144358567
PSAP	Ch10:73588801	NM_002778	c.409C>G	p.L137V	Het	Combined SAP deficiency	AR	Novel variant
MAN1B1	Ch9: 140002934	NM_016219	c.1991C>T	p.T664M	Het	Mental retardation, autosomal recessive 15	AR	Reported in ESP5400 and or Thousand Genomes
NPC1	Chr18:21166261	NM_000271	c.47G>A	p.C16Y	Het	Niemann–Pick disease, type D	AR	Novel variant
BRAT1	Chr7:2579447	NM_152743	c.1471G>A	p.G491S	Het	Rigidity and multifocal seizure syndrome, lethal neonatal	AR	Father also heterozygous
BRAT1	Chr7: 2582935	NM_152743	c.826G>A	p.D276N	Het	Rigidity and multifocal seizure syndrome, lethal neonatal	AR	Mother heterozygous, rs146546197
PEX16	Chr11:45931818	NM_004813	c.995_997delTCT	p.F332del	Hom	Zellweger syndrome, complementation 9	AR	Novel variant, both parents heterozygous
