# Supplementary material for: Dataset for a case report of a homozygous PEX16 F332del mutation
Source: Data Brief. 2015 Dec 17;6:722–7. doi: 10.1016/j.dib.2015.12.011 (PMC4737951; doi:10.1016/j.dib.2015.12.011)
Supplement: Supplementary file 1 — Supplementary material [file mmc1.doc]

Manuscript No.: DIB-D-15-00407
Title: Dataset for a Case Report of a Homozygous PEX16 F332del mutation
Journal Title: Data in Brief
Corresponding Author: Dr. Michael F. Wangler
All Authors: Michael F. Wangler
Submit Date: Oct 06, 2015

Dear DIB,

**The Authors of this manuscript note no conflict of interest**

Michael Wangler, M.D.

Molecular and Human Genetics

Baylor College of Medicine

Houston Texas USA
